# Association of Stress and Neighborhood Social Context With Actigraphy-Measured and Self-Reported Adolescent Sleep Outcomes

**DOI:** 10.1016/j.jadohealth.2026.02.022

**Published:** 2026-05-05

**Authors:** Gabrielle DiFiore, Hannah Martin, Jonathan A. Mitchell, Ian Barnett, Douglas Wiebe, Mathias Basner, Alexander G. Fiks, Karen Glanz, Stephanie L. Mayne

**Affiliations:** a Clinical Futures, PolicyLab, and the Possibilities Project, Children’s Hospital of Philadelphia, Philadelphia, Pennsylvania; b Department of Pediatrics, University of Pennsylvania Perelman School of Medicine, Philadelphia, Pennsylvania; c Division of Gastroenterology, Hepatology, and Nutrition, Children’s Hospital of Philadelphia, Philadelphia, Pennsylvania; d Department of Biostatistics, Epidemiology, and Informatics, University of Pennsylvania Perelman School of Medicine, Philadelphia, Pennsylvania; e Department of Emergency Medicine, University of Michigan, Ann Arbor, Michigan; f Department of Psychiatry, University of Pennsylvania Perelman School of Medicine, Philadelphia, Pennsylvania; g Leonard Davis Institute for Healthcare Economics, University of Pennsylvania Perelman School of Medicine, Philadelphia, Pennsylvania; h School of Nursing, University of Pennsylvania, Philadelphia, Pennsylvania

**Keywords:** Stress, Sleep, Actigraphy, Neighborhood environment, Collective efficacy, Safety

## Abstract

**Purpose:**

Chronic stress and unfavorable neighborhood environments may increase adolescents’ risk for poor sleep. Few studies have examined whether neighborhood social environments moderate associations of adolescents’ perceptions of stress with actigraphy-measured and self-reported sleep outcomes.

**Methods:**

In a cross-sectional study of adolescents aged 15–18 (n = 163) years, perceived stress (10-item Perceived Stress Scale) and perceived neighborhood collective efficacy and safety were assessed via survey. Over 14 days, actigraphy measured nightly sleep duration and timing, while ecological momentary assessment (EMA) measured daily stress, self-reported sleep problems, and sleep environment disruptions. Multivariable mixed effects regression estimated associations of stress and neighborhood social factors with nightly sleep patterns and self-reported sleep outcomes, while multivariable linear regression estimated associations with sleep variability (intraindividual standard deviation of each sleep measure).

**Results:**

Higher 10-item Perceived Stress Scale scores and daily EMA-reported stress were associated with more variable sleep duration, onset, and offset timing, and higher and more variable sleep problem scores. Daily EMA-reported stress was also associated with earlier sleep timing. Higher neighborhood collective efficacy was associated with less variable sleep duration and timing, and lower and less variable sleep problem scores. Neighborhood collective efficacy and safety moderated associations between stress and several sleep outcomes (e.g., stronger associations between stress and sleep variability were present among adolescents with low neighborhood safety or collective efficacy).

**Discussion:**

Lower perceived stress and higher neighborhood collective efficacy were associated with less variable sleep patterns and lower sleep problem scores. Results suggest positive neighborhood social environments may moderate the relationship of stress with adolescent sleep variability.

Sleep is a fundamental pillar of health and development, particularly during adolescence — a period marked by significant biological, psychological, and social changes. [1] Adequate sleep supports memory consolidation, emotional regulation, cognitive functioning, and physical growth, which are crucial for successful adolescent development. [1–4] However, national data show most US adolescents fail to sleep the recommended 8–10 hours per night, with lower sleep durations observed among youth living in disadvantaged contexts. [5,6] While biological factors such as shifts in circadian rhythms play a role in adolescent sleep patterns, [2] mounting evidence highlights the influence of psychosocial and environmental stressors, particularly chronic stress and adverse home or neighborhood conditions. [7–13].

Chronic stress during adolescence can disrupt sleep regulation and contribute to short- and long-term adverse outcomes. [14,15] Stressors such as family conflict, academic pressure, financial strain, or community violence may heighten physiological arousal, dysregulate cortisol rhythms, and increase risk for anxiety and depression. [16–18] Rising rates of adolescent anxiety and depression underscore the importance of understanding how stress and sleep are intertwined. [19,20] Stress-related physiological and emotional responses can interfere with sleep onset, reduce sleep quality, and shorten sleep duration. [8,9,21–23] Stress is also associated with greater night-to-night variability in sleep duration and timing, reflecting how irregular sleep patterns can contribute to emotional well-being and cognitive functioning. [23–26] Despite daily fluctuations in stress, few studies have examined how day-to-day stress relates to adolescent sleep outcomes.

The neighborhood social environment is an understudied context where stress and sleep may intersect. Neighborhoods often reflect structural inequalities, with adolescents in disadvantaged neighborhoods more likely to encounter safety concerns, community disorganization, and low collective efficacy (shared norms and trust among neighbors). [27,28] These neighborhood characteristics shape adolescents’ daily experiences, and emerging research suggests they influence adolescent sleep. [7,8,12,13,27] Living in unsafe neighborhoods might increase hypervigilance or anxiety at bedtime, reduce opportunities for physical activity, or increase time spent in sedentary screen-based activities, all of which could negatively impact sleep. Similarly, low collective efficacy may reduce opportunities for restorative activities and social support that promote healthy sleep. To date, most research has examined neighborhood social environments and stress in isolation, overlooking the complex and potentially compounding nature of these environmental and psychosocial factors. For example, a more supportive neighborhood social environment, with greater perceived safety and collective efficacy, might buffer stress responses and thereby support better sleep outcomes. [7,9–13] Addressing this gap is critical for developing targeted, context-sensitive interventions to promote adolescent sleep health and may inform community and policy efforts aimed at reducing health disparities. By integrating constructs from socioecological models of health with stress physiology, this study aims to improve our understanding of how broader social environments intersect with individual psychological factors that shape adolescent sleep patterns and variability (Figure 1). Our objective was to examine associations of stress and neighborhood social factors (collective efficacy, safety), with sleep outcomes among adolescents, and to determine whether neighborhood collective efficacy and safety moderated associations of stress with sleep outcomes.

## Methods

### Study sample

This study uses data from the Teen Neighborhood Activity Patterns and Sleep study — a cross-sectional observational study of adolescents’ sleep outcomes and their daily environmental exposures at home and within their neighborhoods. The study took place between February 2022 and August 2024 and included adolescents aged 15–18 years who lived and attended school in Philadelphia, Pennsylvania, spoke English and had a smartphone with data and texting capabilities. Adolescents were excluded if they had medical conditions directly related to sleep (e.g., sleep apnea), conditions that would have impacted daily mobility, took medications known to affect sleep or mobility, or if they were experiencing acute illness that could interfere with daily activities. In general, participants were healthy. Adolescents with diagnoses such as anxiety or depression were not excluded, as our aim was to capture a representative sample of adolescents’ lived experiences.

Recruitment occurred through the Children’s Hospital of Philadelphia (CHOP) Recruitment Enhancement Core, which sent email invitations to eligible families receiving primary, specialty, or emergency care in the CHOP network and posted flyers at CHOP primary care locations and on the main hospital campus. Parents and 18-year-old adolescents provided informed consent, while 15–17-year-old adolescents provided assent. Upon completion of an initial survey, each adolescent was mailed a wrist-worn actigraphy device to measure sleep patterns over 14 days and downloaded a smartphone application used for ecological momentary assessment (EMA) surveys. Study procedures were approved by the CHOP Institutional Review Board.

### Dependent variables

Dependent variables of interest included (1) actigraphy-measured sleep patterns (duration, onset, offset timing) and (2) self-reported sleep problems and sleep environment disruptions. To measure adolescent sleep patterns, we used GENEActiv Original devices (Activinsights Ltd.), which are wrist-worn tri-axial accelerometers. Participants were instructed to wear the device on their nondominant wrist continuously over the 14-day data collection period. These devices recorded raw accelerometry data at 50 Hz and were returned to the study team by mail. Data were analyzed using the open-source R package “GGIR” which uses raw accelerometry data collected by actigraphy devices to identify sleep and wake periods and generate sleep metrics. We utilized the Cole-Kripke algorithm within GGIR to estimate sleep metrics at the night-level (up to 14 nights per participant), informed by a recent validation study demonstrating that the Cole-Kripke algorithm provides high accuracy against polysomnography when implemented in GGIR. [29] Preliminary quality checks flagged nights based on the following criteria: more than 30% nonwear time, over 30% of the night deemed invalid, sleep duration under 2 hours, wake times before 1:00 a.m., or 100% sleep efficiency. Flagged nights underwent manual review by trained team members (G.D., S.M.) to assess data integrity to determine whether nightly data quality was sufficient for inclusion for analysis.

Actigraphy-derived sleep outcomes included total nightly sleep duration (measured as total hours classified as sleep occurring between sleep onset and offset within the primary overnight sleep period) and sleep onset and offset timing (measured as hours from midnight, e.g., −1.0 = 23:00 and 6.5 = 06:30) on each night. As secondary outcomes, we examined sleep efficiency (percentage of time spent asleep within the sleep period) and wake after sleep onset (WASO; time in minutes spent awake during the sleep period).

Self-reported sleep outcomes were assessed through daily EMA surveys administered using the LifeData platform (lifedatacorp.com). Adolescents received prompts to complete brief surveys four times per day during the 14-day data collection period, which captured in-the-moment data on their physical surroundings, stress, and health behaviors. Surveys were preprogrammed to be sent to participants’ phones during four main timeframes across the day, further randomized within each window to introduce variability and reduce response bias. On weekdays, morning surveys were sent between 6 and 8 am (prior to school hours), while weekend morning surveys were sent between 10 am and 12 p.m. Early afternoon surveys were sent between 2 and 4 pm, late afternoon surveys between 5 and 7 pm, and evening surveys between 8 and 10 p.m. Items that assessed sleep environment or sleep problems were included only on the morning survey and referred to the prior night.

Self-reported sleep problems were assessed using four items adapted from the Patient Reported Outcome Measurement Information System Sleep Disturbance scale, which asked about problems related to subjective sleep quality such as difficulty falling asleep, staying asleep, and perceptions of sleep quality (see Table S1). Responses were combined into a summary score for each day within the study period, ranging from 0 to 4 (α = .78), with higher scores demonstrating more sleep problems reported on a given night. Perceptions of disruptions in the home sleep environment (e.g., uncomfortable bed, excessive noise) were assessed with eight items, which were previously used with adolescent populations. [30,31] Similarly, we combined responses into a summary score for each day within the study period, ranging from 0 to 8 (α = .48) with higher scores demonstrating more sleep environment disruptions reported on a given night.

All actigraphy-measured and self-reported sleep outcomes were treated as continuous variables. Nights were categorized as “school nights” (Sunday through Thursday) or “weekend nights” (Friday and Saturday), with nights preceding federal holidays considered weekend night. In addition to nightly sleep outcomes, we also examined within-participant variability in each sleep outcome by calculating person-specific standard deviations (SDs) for each sleep metric across nights.

### Independent variables

Our primary exposure of interest was adolescents’ perceived stress, which was assessed in two ways — once on the initial survey and repeatedly via daily EMA surveys. Adolescents first completed the validated, 10-item Perceived Stress Scale (PSS-10), [32] one of the most widely used measures of self-reported perceived stress. Item responses were summed and analyzed as a continuous variable ranging from 0 to 40 (α = .87). In addition, the EMA surveys assessed daily stress using three items. The first question assessed current stress level by asking: “How stressed do you feel right now?”, with response options as follows: not at all stressed, a little stressed, moderately stressed, quite a bit stressed, and extremely stressed. Responses were coded numerically from 1 to 5 and averaged together to create an overall daily continuous stress score. The second question asked: “Over the past 2 hours, has anything stressful happened to you?”. If participants responded with “yes,” they were able to indicate which option(s) had caused them stress from the following list: school, job, family conflict, friend conflict, social media, worries about the future, not having enough money to buy the things you need, feeling unsafe, and something else. The second question was coded as a binary variable indicating whether any stressor was reported on a given day. Daily EMA stress variables were then aligned with sleep outcomes from the subsequent night (i.e., the night directly following the stress report) to examine within-person daily-level associations between stress and sleep.

### Moderators

Neighborhood collective efficacy and safety were assessed on the one-time adolescent survey. Neighborhood collective efficacy was assessed using the 10-item Collective Efficacy scale from the Project on Human Development in Chicago Neighborhoods study [33], which included five items about social cohesion (reflecting connectedness between neighbors) and five items about informal social control (neighbors’ willingness to intervene in challenging situations, Table S1). Neighborhood safety was assessed using a two-item measure with high internal consistency and test-retest reliability among urban adults. [34–37] For both neighborhood social measures, Likert-scaled responses to each item were averaged to create a continuous score ranging from 1 to 5, with higher scores indicating more favorable neighborhood perceptions (α = .89 for collective efficacy and α = .76 for safety).

### Covariates

Covariates included potential demographic and socioeconomic confounders identified a priori that we hypothesized to be associated with stress, neighborhood social factors, and sleep health. Demographic covariates were self-reported by adolescents and included age, sex at birth, race, and ethnicity. Socioeconomic covariates were self-reported by parents, which included parent educational attainment, household income, and marital status. We also included nightly indicators for school night status (school night vs. weekend night) and indicators for month, to account for seasonal variation in sleep due to factors such as sunrise/sunset timing, school versus summer vacation, and broader seasonal differences.

### Statistical analysis

The analysis included adolescents who had complete data from the adolescent baseline survey on the covariates listed above, PSS-10 scores, and neighborhood social environment measures, and at least one night of actigraphy data or self-reported sleep data (n = 163). Of those 163 participants, 152 had valid actigraphy data (1,900 nights in total) and 158 had self-reported sleep outcomes measured by EMA surveys (1,681 nights in total). Daily measures of stress, reported via EMA, were available from 153 participants (1,907 days in total). Both actigraphy-measured and self-reported sleep outcomes were measured on a nightly-basis and described at the night-level throughout the analysis, except for the variability measures (intraindividual SDs), which were calculated at the participant level. Daily stress was analyzed at the day-level (e.g., daily average EMA-reported stress score). To examine the associations of daily stress with sleep variability, daily stress measures were aggregated to the person-level by averaging across days.

The distribution of demographic characteristics, perceived stress, and neighborhood social environment measures across adolescents, and of sleep outcomes across nights, were examined using descriptive statistics. To determine associations of stress and neighborhood social factors with nightly sleep outcomes, we used linear mixed effects modeling with random intercepts to account for clustering of multiple days (up to 14) per adolescent. Missingness in our outcome data was accounted for by maximum likelihood estimation. We used separate models to estimate associations of PSS-10 scores, day-level EMA stress measures, neighborhood collective efficacy, and neighborhood safety with each actigraphy-measured or self-reported sleep outcome. All models were adjusted for the covariates listed above. To assess associations with within-person variability in sleep outcomes, we conducted multivariable linear regression analyses using person-level outcomes (i.e., intraindividual SDs for each sleep outcome parameter). These models included the same covariates as described above, excluding school night status. Variability models were limited to 148 participants who had at least two nights of valid actigraphy data, the minimum required to calculate variability estimates. As a sensitivity analysis, we repeated the models above among a subset of adolescents who had at least five nights of valid actigraphy data (n = 136), as prior research indicates this may improve the reliability of actigraphy-derived sleep assessment. [38].

To explore whether the neighborhood social environment moderated associations between stress and sleep outcomes, we tested interaction effects of PSS-10 score, EMA continuous daily stress score, and presence of daily stressors with neighborhood collective efficacy and safety in separate models, adjusted for covariates as described above. Analyses were completed in R (version 4.4.0; Vienna, Austria) and Stata (version 18.0; Stata-Corp, College Station, TX).

## Results

Among 163 adolescents included in this analysis, the mean age was 16.2 years (SD: .87), and 58.9% were female (Table 1). More than half (50.9%) identified as non-Hispanic Black, 28.2% as non-Hispanic White, 12.3% as Hispanic or Latino, and 8.6% reported multiple races or another category. Forty point five percent of households reported an annual income of $100,000 or more, and 30.1% of parents held a graduate or professional degree. The mean Perceived Stress Scale (PSS-10) score was 18.51 (SD: 7.39), measured from 0 to 40. The average neighborhood collective efficacy score was 3.24 (SD: .75) and the average neighborhood safety score was 3.27 (SD: 1.07) on a 1–5 scale. The average nightly sleep duration was 6.5 hours (SD: 1.8). Correlations between neighborhood environment and stress measures are presented in Table S2.

### Associations with actigraphy-measured sleep outcomes

Table 2 shows associations between actigraphy-measured sleep outcomes and the independent variables (perceived and daily stress) and moderator variables (neighborhood safety and collective efficacy). Higher PSS-10 scores were associated with greater variability in sleep duration (β: .03, 95% confidence interval [CI]: .01, .05; i.e., a 1.8-minute higher sleep duration SD for each unit increase of PSS-10 score), onset (β: .04, 95% CI: .01, .06), and offset timing (β: .03, 95% CI: .002, .05) within participants but not with overall levels of sleep duration or timing. Higher PSS-10 scores were also associated with more variable sleep efficiency and WASO (Table S3). Higher daily stress scores, reported via EMA, were associated with earlier sleep onset (β: −.22, 95% CI: −.42, −.02), earlier sleep offset (β: −.30, 95% CI: −.52, −.07), and more variable sleep duration and offset timing (Table 2). Reporting any stressor on a given day was associated with more variable sleep duration, onset, and offset timing and with more variable WASO (Table S3) but not with overall levels of sleep duration or timing.

Higher neighborhood collective efficacy was associated with lower variability in sleep duration (β: −.27, 95% CI: −.46, −.08), onset (β: −.35, 95% CI: −.56, −.13), and offset timing (β: −.32, 95% CI: −.53, −.11). Neighborhood safety was associated with slightly lower sleep onset variability, although the CI crossed the null (Table 2). Neither neighborhood collective efficacy nor safety was associated with overall levels of sleep duration or timing, nor with sleep efficiency or WASO (Table S3).

In sensitivity analyses restricted to adolescents with at least five nights of actigraphy data (n = 136), results were largely consistent with the full sample, with minimal changes in between-person associations but slightly larger changes for the within-person sleep variability measures (Table S4).

### Self-reported sleep outcomes

Table 3 shows the associations between self-reported sleep outcomes and stress and neighborhood social environment measures. Higher PSS-10 score was associated with higher and more variable self-reported sleep problem and sleep environment disruption scores, while daily stress score was associated with higher self-reported sleep problem scores and greater variability in sleep environment disruptions. Reporting any stressor on a given day was not associated with overall sleep problem or sleep environment scores but was associated with greater variability in sleep problem and sleep environment disruption scores across nights.

Higher neighborhood collective efficacy was associated with lower self-reported sleep problem scores, and with less variable sleep problem and sleep environment disruption scores (Table 3). Neighborhood safety was not associated with self-reported sleep problems or environmental disruptions, but participants who reported higher perceived safety had lower variability in self-reported sleep problem scores across nights (Table 3).

### Moderation analysis

**Figure 2.**
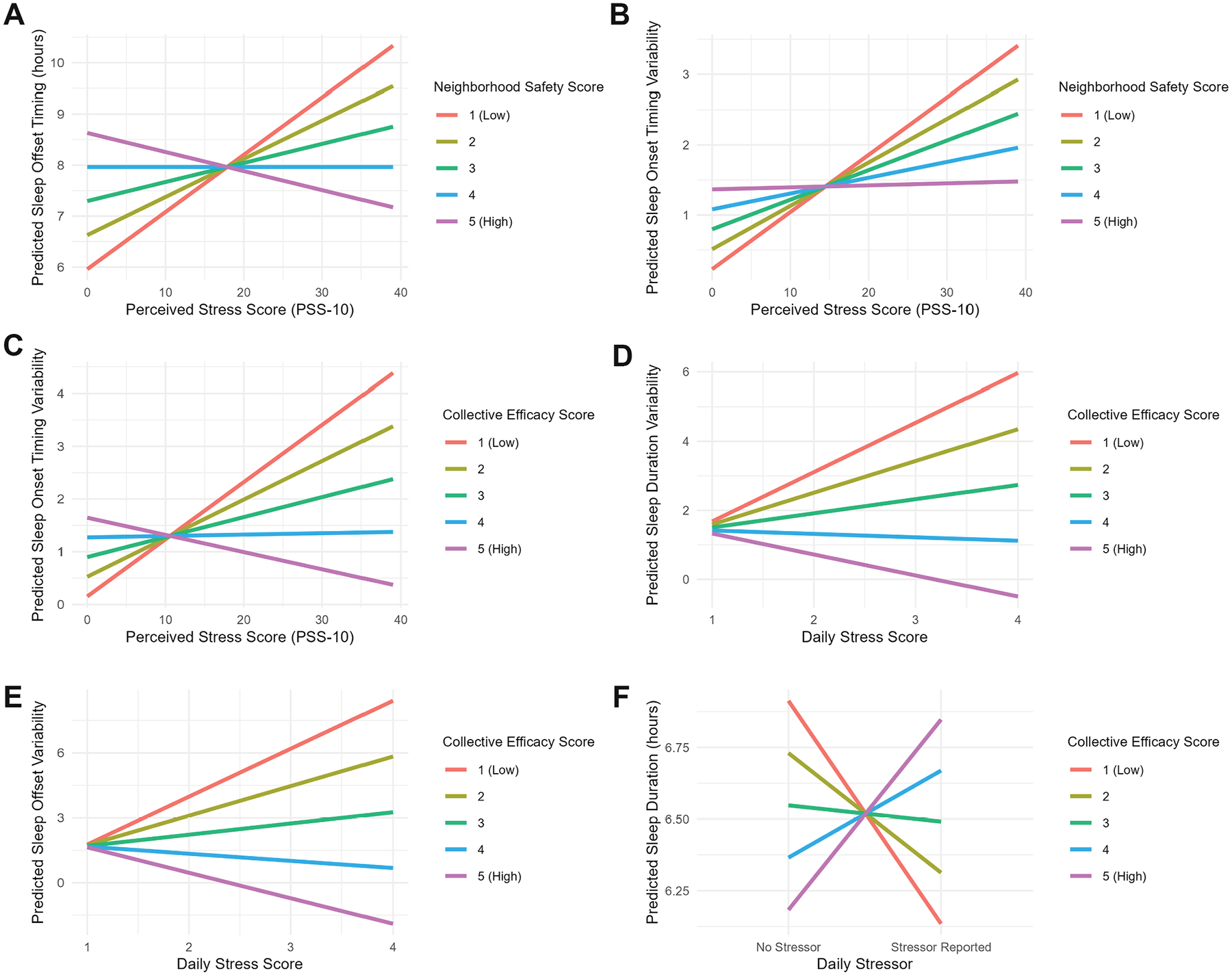
Interactions between stress and neighborhood social environment variables. Figures present results from models with significant interaction terms (*p* < 0.05) between perceived stress variables (10-item Perceived Stress Scale [A–C], ecological momentary assessment-based averaged daily stress score [D–E] and daily stressor reported [F]) and neighborhood safety or collective efficacy.

There was evidence that neighborhood social environment exposures moderated associations of stress with several sleep outcomes. Out of 60 total interactions tested, only six were statistically significant (Tables S5 and S6). Figure 2 plots these interactions. Two interactions were present for neighborhood safety and PSS-10 scores (Figure 2A, B), while four involved neighborhood collective efficacy and a mix of PSS-10 scores and daily stress measures (Figure 2C–F). In general, these patterns show stronger associations of stress with more adverse sleep outcomes among adolescents with the lowest levels of perceived neighborhood safety and collective efficacy. No significant interactions were found for self-reported sleep outcomes (Table S5).

## Discussion

This study examined associations of adolescents’ stress, perceptions of their neighborhood social environment, and sleep outcomes measured using actigraphy and self-report. We found that higher perceived stress, as measured by the PSS-10, and daily stress, measured via EMA, were associated with greater variability in sleep duration and timing, as well as higher self-reported sleep problem scores, suggesting stress may influence the consistency and subjective quality of sleep. In addition, we found neighborhood collective efficacy was associated with less variability in sleep duration, onset, and offset timing, as well as lower levels of self-reported sleep problems, suggesting that neighborhood-level social support may promote greater sleep regularity. Lastly, our moderation analyses suggest that a more positive neighborhood social environment might buffer the relationship between stress and certain sleep outcomes, although interactions were only detected for a small number of associations.

Our results support prior evidence linking stress to adolescent sleep problems. Chronic and daily stress impairs emotional regulation, increases arousal, and disrupts circadian rhythms, contributing to sleep difficulties. [14,39,40] The associations we observed between higher PSS-10 scores and increased sleep problems and environmental disruptions are consistent with literature emphasizing the role of stress in shaping adolescents’ sleep experiences. [16–18] Interestingly, while higher PSS-10 scores were associated with more self-reported sleep disturbances, it was not related to actigraphy-based sleep duration and timing, while daily EMA-reported stress was associated with differences in actigraphy-based sleep timing. These findings reinforce that objective and self-reported sleep outcomes reflect different but complementary dimensions of sleep health. Furthermore, higher stress was associated with greater night-to-night variability in sleep duration and timing, suggesting stress may affect both perceived sleep quality and regularity of sleep patterns. Prior studies suggest that irregular sleep patterns are associated with poorer emotional regulation, increased risk for anxiety and depression, and less desirable academic functioning, highlighting the importance of assessing variability as a distinct dimension of sleep health. [24,41,42].

In addition, findings add to a growing body of research on how perceived neighborhood characteristics relate to adolescent sleep. [8,9,27] Prior studies found that living in a neighborhood with greater disadvantage, greater disorder and lower social cohesion are associated with poorer sleep. [7,12,13] We found that higher neighborhood collective efficacy was associated with more consistent sleep patterns across nights, including less variability in sleep duration and timing — suggesting adolescents in more cohesive neighborhoods may benefit from more stable sleep schedules. Neighborhood collective efficacy might impact sleep regularity by enhancing social support and reinforcing social norms toward sleep-promoting behaviors [43,44] (e.g., a regular bedtime), [7,43] while less cohesive neighborhoods may heighten vigilance or social isolation, adversely affecting sleep through dysregulation of the hypothalamic-pituitary-adrenal axis. [45] Limited associations with perceived neighborhood safety were surprising given prior findings in adolescent populations, [46] though some studies suggest stronger associations for other aspects of the neighborhood social environment (e.g., social cohesion) and sleep outcomes than for safety. [9,43] In addition, the findings of our moderation analysis suggest adverse associations of stress with sleep variability are stronger among adolescents with the lowest perceived neighborhood safety and collective efficacy, although interactions were not consistently observed across all stress-neighborhood measure combinations.

This study has several limitations. First, stress and neighborhood measures relied on self-report, which may be subject to bias. While self-report captures important perceptions influencing health behaviors, future research could incorporate objective or observational measures of neighborhood conditions. Household stress was not assessed though it may be an important factor linked to adolescent mental health and sleep outcomes. While we assessed adolescents’ perceptions of nighttime sleep disruptions within the home, we did not assess neighborhood-level disruptions (e.g., noise, traffic, and light pollution). Second, our study was conducted in a single US city, limiting generalizability to other regions or rural communities. Our sample was predominantly of higher socioeconomic status than the overall population of Philadelphia (median income: $60,000), and may not represent adolescents from lower socioeconomic status backgrounds who often face different stressors and sleep challenges. Additionally, the 14-day data collection period may not fully reflect longer-term patterns. Also, some EMA surveys may have overlapped with school hours or activities (e.g., sports practice), potentially affecting response rates. The cross-sectional study design also limits causal inference. Future longitudinal work is needed to determine temporal relationships and mechanisms, such as stress and health behaviors (e.g., physical activity, screen time, diet) as mediators in the relationship of collective efficacy with sleep variability and self-reported sleep quality. Finally, while our study was powered to detect moderate associations of neighborhood environmental exposures with adolescent sleep outcomes, larger studies may be better positioned to detect smaller magnitudes of interactions between neighborhood factors and stress.

### Conclusions

Adolescents’ stress and perceptions of their neighborhood environments may play a role in the consistency of their sleep patterns across nights and in how they experience and report sleep problems. Our findings highlight that incorporating both self-reported sleep assessments and sleep variability metrics provides a more complete picture of the relationship of environmental and psychological factors with adolescent sleep.

## Supplementary Material

1

Supplementary data related to this article can be found at http://doi:10.1016/j.jadohealth.2026.02.022.

## Figures and Tables

**Figure 1.**
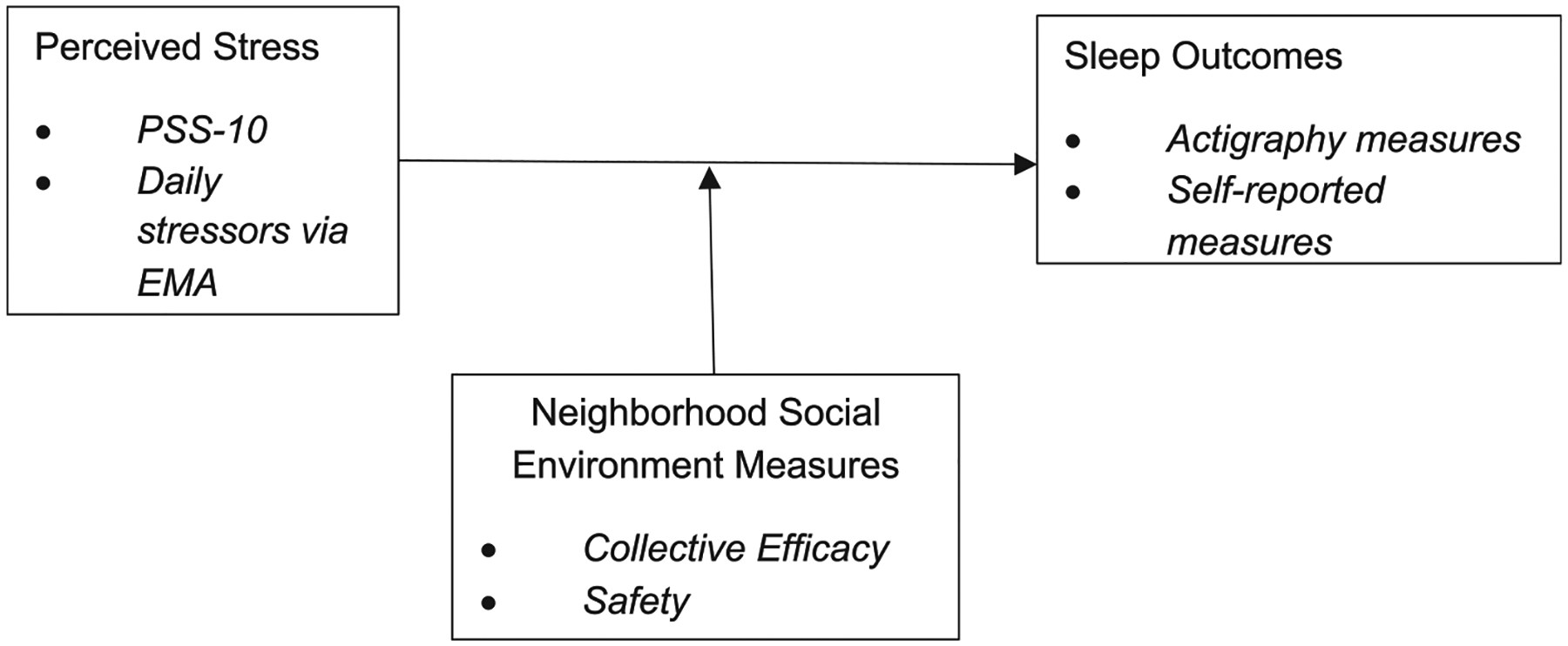
Conceptual model of hypothesized relationships between stress, neighborhood social environment, and adolescent sleep outcomes.

**Table 1:** Participant demographics (n = 163) and summary of sleep outcomes and daily stress across participants

Characteristic	N (%)
Sociodemographics	
Age (mean ± SD)	16.2 ± .87
Sex	
Female	96 (58.9%)
Male	67 (41.1%)
Race	
Non-Hispanic White	46 (28.2%)
Non-Hispanic Black	83 (50.9%)
Hispanic or Latino	20 (12.3%)
Other or multiple races	14 (8.6%)
Household income^a^	
Less than $25,000	22 (13.5%)
$25,000 to $49,999	28 (17.2%)
$50,000 to $99,999	47 (28.8%)
$100,000 or more	66 (40.5%)
Parent education level^a^	
High school degree or less	25 (15.3%)
Some college	55 (33.7%)
Bachelor’s degree	34 (20.9%)
Graduate or professional degree	49 (30.1%)
Parent marital status^a^	
Married/living with partner	80 (49.1%)
Not married/living with partner	83 (50.9%)
	Mean ± SD
Measures of perceived stress	
Perceived Stress Scale (range: 0–40)	18.51 ± 7.39
Daily reported stress score (range: 1–5)^b^	1.48 ± .68
Specific daily stressors, n (%) of days^b^	
Any stressor reported	582 (24.8%)
School	257 (44.2%)
Family conflict	121 (20.8%)
Friend conflict	121 (20.8%)
Worry about the future	73 (12.5%)
Other^c^	299 (51.4%)
Neighborhood social environment	
Collective efficacy (range: 1–5)	3.24 ± .75
Perceived safety (range: 1–5)	3.27 ± 1.07
Actigraphy-measured sleep outcomes^d^	
Sleep duration (hours)	6.5 ± 1.8
Sleep onset (hours from 00:00)	.3 ± 2.2
Sleep offset (hours from 00:00)	8.3 ± 2.5
Sleep efficiency (percentage)	82.6 ± 9.2
Wake after sleep onset (minutes)	86.2 ± 55.5
SD sleep duration (hours)	1.62 ± .74
SD sleep onset (hours from 00:00)	1.63 ± .97
SD sleep offset (hours from 00:00)	1.88 ± .93
Self-reported sleep outcomes^e^	
Sleep problem score (range: 0–4)	.60 (1.10)
Sleep environment disruption score (range: 0–8)	.30 (.70)

a Parent-reported metric.

b Responses to daily stress scale and specific daily stressors were available for 1,907 days from 153 adolescents.

c “Other” stressors included money, job, feeling unsafe, social media, being treated unfairly due to race or gender, and “something else”. Responses to the “something else” choice were a binary (yes/no) option indicating the presence of a stressor not listed among the predefined categories; qualitative details were not collected, so these responses could not be further categorized.

d Actigraphy-measured sleep outcomes were available for 1,900 nights from 152 adolescents.

e Responses to the self-reported sleep problems scale were available for 1,681 nights from 158 adolescents and responses to the home sleep environment disruptions scale were available for 1,691 nights from 159 adolescents.

**Table 2:** Association of stress and neighborhood social environment with actigraphy-assessed adolescent sleep patterns

	Sleep patterns, β (95% CI)^a,b^			Within-person sleep variability, β (95% CI)^b,c^		
	Duration	Sleep onset	Sleep offset	Duration SD	Sleep onset SD	Sleep offset SD
Stress						
Perceived Stress Scale (PSS-10)	.01 (−.02, .03)	.01 (−.03, .05)	.03 (−.01, .07)	.03 (.01, .05)*	.04 (.01, .06)*	.03 (.002, .05)*
Daily stress score	−.04 (−.21, .14)	−.22 (−.42, −.02)*	−.30 (−.52, −.07)*	.32 (.02, .62)*	.29 (−.05, .64)	.35 (.01, .68)*
Daily stressor reported	.03 (−.19, .25)	−.18 (−.41, .05)	−.10 (−.36, .17)	.82 (.17, 1.47)*	.83 (.08, 1.58)*	1.30 (.60, 2.01)*
Neighborhood social environment						
Collective efficacy	−.07 (−.28, .15)	.10 (−.24, .45)	.02 (−.34, .39)	−.27 (−.46, −.08)*	−.35 (−.56, −.13)*	−.32 (−.53, −.11)*
Safety	−.02 (−.17, .13)	−.01 (−.25, .24)	−.07 (−.33, .18)	.02 (−.12, .15)	−.12 (−.28, .03)	−.06 (−.22, .09)

SD = standard deviation; CI = confidence interval.

a Estimated using mixed effects linear regression models with participant random intercepts, adjusting for participant age, sex, race/ethnicity, household income, parental education, parental marital status, school night status, and month.

b Actigraphy-measured sleep outcomes were available for 152 adolescents.

c Estimated using general linear regression models, adjusting for participant age, sex, race/ethnicity, household income, parental education, parental marital status, and month. Because sleep variability measures were calculated at the participant level (i.e., by taking the person-specific standard deviation of each sleep variable across nights), models were at the participant rather than night level. Models included 148 participants with ≥2 nights of actigraphy data (needed to calculate variability measures).

* *p* value <.05.

**Table 3:** Association of stress and neighborhood social environment with self-reported adolescent sleep outcomes

	Sleep outcome scores, β (95% CI)^a,b^		Within-person variability, β (95% CI)^a,c^	
	Sleep problems	Sleep environment disruptions	Sleep problems SD	Sleep environment disruptions SD
Stress				
Perceived Stress Scale (PSS-10)	.03 (.01, .05)*	.02 (.01, .03)*	.02 (.01, .04)*	.01 (.004, .02)*
Daily stress score	.11 (.01, .20)*	.03 (−.03, .09)	.15 (−.06, .36)	.26 (.12, .40)*
Daily stress reported	.07 (−.04, .19)	−.01 (−.08, .06)	1.05 (.62, 1.47)*	.36 (.04, .68)*
Neighborhood social environment				
Collective efficacy	−.17 (−.33, −.02)*	−.09 (−.20, .02)	−.14 (−.27, −.01)*	−.10 (−.19, −.002)*
Safety	−.09 (−.20, .01)	−.03 (−.11, .05)	−.12 (−.21, −.02)*	−.03 (−.10, .04)

SD = standard deviation; CI = confidence interval.

a Estimated using mixed effects linear regression models with participant random intercepts, adjusting for participant age, sex, race/ethnicity, household income, parental education, parental marital status, school night status, and month.

b Self-reported sleep outcomes were available for 159 adolescents.

c Estimated using general linear regression models, adjusting for participant age, sex, race/ethnicity, household income, parental education, parental marital status, and month. Because sleep variability measures were calculated at the participant level (i.e., by taking the person-specific standard deviation of each sleep variable across nights), models were at the participant rather than night level. Models included 148 participants with ≥2 nights of actigraphy data (needed to calculate variability measures).

* *p* value <.05.
