# Supplementary material for: Association of Stress and Neighborhood Social Context With Actigraphy-Measured and Self-Reported Adolescent Sleep Outcomes
Source: J Adolesc Health. Author manuscript; Available in PMC 2026 Jul 6. (PMC13334496; doi:10.1016/j.jadohealth.2026.02.022)
Supplement: 1 [file NIHMS2191807-supplement-1.docx]

**Supplemental Table 1.** **Self-Reported Measures of Sleep and Neighborhood Environment**

|  | **Mode of Assessment** | **Question:** | **Response Options** | **Parameterization** |
| --- | --- | --- | --- | --- |
| Sleep Problems^1^ | Daily EMA^5^ | I had difficulty falling asleep | Yes, No | Any problem (selecting “yes” for any item) versus none (selecting “no” for each) |
|  |  | I slept through the night |  |  |
|  |  | I had a problem with my sleep |  |  |
|  |  | I had trouble sleeping |  |  |
| Sleep Environment Disruptions^2^ | Daily EMA^5^ | Which of the following kept you from sleeping well last night? (1) Noise outside, (2) noise inside, (3) someone snoring, (4) uncomfortable bed, (5) temperature of the room, (6) TV, radio, or computer, (7) Phone or tablet, (8) too much light, (9) none of the above | Check all that apply | Any sleep disruption (checking any of responses 1-8) versus none (checking “none of the above”) |
| Neighborhood Collective Efficacy^3^ | One-time survey | People around here are willing to help their neighbors. | Strongly disagree (1); Disagree (2); Neither agree nor disagree (3); Agree (4); Strongly agree (5) | Aggregated into a summary score by averaging each item (possible range: 1-5) with a higher score reflecting greater neighborhood collective efficacy |
|  |  | This is a close-knit neighborhood. |  |  |
|  |  | People in this neighborhood can be trusted. |  |  |
|  |  | People in this neighborhood generally don't get along with each other. |  |  |
|  |  | People in this neighborhood do not share the same values. |  |  |
|  |  | Neighbors can be counted on to intervene if children were skipping school and hanging out on a street corner. |  |  |
|  |  | Neighbors can be counted on to intervene if children were spray-painting graffiti on a local building. |  |  |
|  |  | Neighbors can be counted on to intervene if children were showing disrespect to an adult. |  |  |
|  |  | Neighbors can be counted on to intervene if a fight broke out in front of their house. |  |  |
|  |  | Neighbors can be counted on to intervene if the fire station closest to their home was threatened with budget cuts. |  |  |
| Perceived Neighborhood Safety^4^ | One-time survey | I feel safe walking in my neighborhood, day or night | Strongly disagree (1); Disagree (2); Neither agree nor disagree (3); Agree (4); Strongly agree (5) | Aggregated into a summary score by averaging each item (possible range: 1-5) with a higher score reflecting greater perceived neighborhood safety |
|  |  | Violence is not a problem in my neighborhood |  |  |

^1^Adapted from Forrest CB, Meltzer LJ, Marcus CL, et al. Development and validation of the PROMIS Pediatric Sleep Disturbance and Sleep-Related Impairment item banks. *Sleep*. Jun 1 2018;41(6)

^2^Adapted from Bagley EJ, Kelly RJ, Buckhalt JA, El-Sheikh M. What keeps low-SES children from sleeping well: the role of presleep worries and sleep environment. *Sleep Med*. Apr 2015;16(4):496-502

^3^Sampson RJ, Raudenbush SW, Earls F. Neighborhoods and Violent Crime: A Multilevel Study of Collective Efficacy. Science. 1997;277(5328):918-924. doi:10.1126/science.277.5328.918

^4^Echeverria SE, Diez-Roux AV, Link BG. Reliability of self-reported neighborhood characteristics. J Urban Health. 2004;81(4):682-701. doi:10.1093/jurban/jth151

^5^Ecological Momentary Assessment (EMA) surveys administered using the LifeData platform (lifedatacorp.com) where adolescents received prompts to complete brief surveys four times per day during the 14-day data collection period.

**Supplemental Table 2. Spearman Correlation Coefficients Between Stress and Neighborhood Social Environment Variables**

|  | Perceived Stress Scale (PSS-10) | Daily Stress Score | Daily Stress Reported | Collective Efficacy | Safety |
| --- | --- | --- | --- | --- | --- |
| Perceived Stress Scale (PSS-10) | 1.00 | 0.32 | 0.22 | -0.25 | -0.28 |
| Daily Stress Score | 0.32 | 1.00 | 0.50 | -0.02 | -0.03 |
| Daily Stress Reported | 0.22 | 0.50 | 1.00 | -0.08 | -0.09 |
| Collective Efficacy | -0.25 | -0.02 | -0.08 | 1.00 | 0.57 |
| Safety | -0.28 | -0.03 | -0.09 | 0.57 | 1.00 |

**Supplemental Table 3. Association of Stress and Neighborhood Social Environment with Actigraphy-Assessed Sleep Quality**

|  | Sleep Quality Metrics,  β (95% CI)^1,2^ | | Within-Person Sleep Variability,  β (95% CI)^1,3^ | |
| --- | --- | --- | --- | --- |
|  | Sleep Efficiency^4^ | WASO^5^ | Sleep Efficiency SD | WASO SD |
| *Stress* |  |  |  |  |
| Perceived Stress Scale (PSS-10) | -0.05 (-0.20, 0.09) | 0.41 (-0.49, 1.31) | 0.08 (0.01, 0.16)* | 0.91 (0.30, 1.52)* |
| Daily stress score | -0.21 (-1.06, 0.64) | 2.00 (-3.38, 7.39) | 0.82 (-0.24, 1.88) | 12.16 (3.46, 20.86)* |
| Daily stress reported | -0.59 (-1.59, 0.42) | 3.07 (-3.37, 9.50) | 1.75 (-0.57, 4.08) | 18.46 (-0.97, 37.90) |
| *Neighborhood Social Environment* |  |  |  |  |
| Collective efficacy | -0.65 (-1.98, 0.67) | 1.43 (-6.82, 9.67) | -0.41 (-1.10, 0.27) | -3.08 (-8.87, 2.70) |
| Safety | -0.06 (-0.87, 0.99) | -0.80 (-6.56, 4.96) | -0.23 (-0.72, 0.25) | -0.05 (-4.16, 4.06) |

SD: Standard Deviation

^1^Estimated using mixed effects linear regression models with participant random intercepts, adjusting for participant age, sex, race/ethnicity, household income, parental education, parental marital status, school night status, and month

^2^Actigraphy-measured sleep outcomes were available for 152 adolescents

^3^Estimated using general linear regression models, adjusting for participant age, sex, race/ethnicity, household income, parental education, parental marital status, and month. Because sleep variability measures were calculated at the participant level (i.e., by taking the person-specific standard deviation of each sleep variable across nights), models were at the participant rather than night level. Models included 148 participants with ≥2 nights of actigraphy data (needed to calculate variability measures).

^4^Sleep efficiency measured as the percentage of time spent asleep within the sleep period.

^5^Wake After Sleep Onset (WASO) measured in minutes as the time in minutes spent awake during the sleep period.

**Supplemental Table 4. Sensitivity Analysis of Adolescents with 5+ nights of Actigraphy Data (N=139)**

|  | Sleep Patterns, β (95% CI)^1,2^ | | | Within-Person Sleep Variability, β (95% CI)^2,3^ | | |
| --- | --- | --- | --- | --- | --- | --- |
|  | Duration | Sleep Onset | Sleep Offset | Duration SD | Sleep Onset SD | Sleep Offset SD |
| *Stress* |  |  |  |  |  |  |
| Perceived Stress Scale (PSS-10) | 0.01 (-0.02, 0.03) | -0.002 (-0.04, 0.04) | 0.02 (-0.02, 0.06) | 0.03 (0.01, 0.05)* | 0.04 (0.02, 0.06)* | 0.02 (-0.001, 0.05) |
| Daily stress score | -0.03 (-0.21, 0.14) | -0.22 (-0.42, -0.02)* | -0.31 (-0.53, -0.08)* | 0.36 (0.08, 0.64)* | 0.31 (-0.03, 0.65) | 0.32 (-0.01, 0.66) |
| Daily stress reported | -0.01 (-0.23, 0.22) | -0.17 (-0.40, 0.07) | -0.12 (-0.39, 0.15) | 0.75 (0.10, 1.39)* | 0.67 (-0.09, 1.44) | 1.12 (0.38, 1.85)* |
| *Neighborhood Social Environment* |  |  |  |  |  |  |
| Collective efficacy | -0.08 (-0.31, 0.14) | 0.20 (-0.16, 0.57) | 0.09 (-0.29, 0.47) | -0.34 (-0.54, -0.15)* | -0.41 (-0.64, -0.19)* | -0.35 (-0.58, -0.13)* |
| Safety | -0.05 (-0.20, 0.10) | 0.06 (-0.19, 0.31) | -0.07 (-0.34, 0.19) | -0.04 (-0.17, 0.10) | -0.18 (-0.34, -0.02)* | -0.08 (-0.24, 0.08) |

SD: Standard Deviation

^1^Estimated using mixed effects linear regression models with participant random intercepts, adjusting for participant age, sex, race/ethnicity, household income, parental education, parental marital status, school night status, and month

^2^Estimated using general linear regression models, adjusting for participant age, sex, race/ethnicity, household income, parental education, parental marital status, and month. Because sleep variability measures were calculated at the participant level (i.e., by taking the person-specific standard deviation of each sleep variable across nights), models were at the participant rather than night level.

*p-value <0.05

**Supplemental Table 5. Interactions of Stress and Neighborhood Social Environment Variables on Actigraphy-Assessed Adolescent Sleep Patterns**

| Interaction Term | Sleep Patterns, β (95% CI)^1^ | | | Within-Person Variability, β (95% CI)^1^ | | |
| --- | --- | --- | --- | --- | --- | --- |
|  | Duration | Sleep Onset | Sleep Offset | Duration SD | Sleep Onset SD | Sleep Offset SD |
| PSS-10 Score x Safety | -0.01 (-0.03, 0.01) | -0.02 (-0.06, 0.01) | -0.04 (-0.07, -0.01)* | -0.01 (-0.02, 0.01) | -0.02 (-0.04, -0.0001)* | -0.0003 (-0.02, 0.02) |
| PSS-10 Score x Collective eff. | 0.001 (-0.03, 0.03) | 0.01 (-0.03, 0.05) | 0.02 (-0.02, 0.06) | -0.01 (-0.03, 0.01) | -0.04 (-0.06, -0.01)* | 0.005 (-0.02, 0.03) |
| Daily stress score x Safety | -0.07 (-0.24, 0.09) | 0.01 (-0.18, 0.19) | -0.10 (-0.30, 0.11) | -0.07 (-0.36, 0.23) | -0.36 (-0.93, 0.20) | -0.18 (-0.51, 0.15) |
| Daily stress score x Collective eff. | 0.18 (-0.08, 0.44) | -0.17 (-0.47, 0.13) | -0.09 (-0.42, 0.25) | -0.50 (-0.99, -0.02)* | -0.36 (-0.93, 0.20) | -0.84 (-1.37, -0.31)* |
| Daily stress x Safety | -0.01 (-0.22, 0.19) | -0.07 (-0.29, 0.14) | -0.12 (-0.36, 0.13) | 0.28 (-0.27, 0.84) | 0.05 (-0.59, 0.69) | 0.50 (-0.10, 1.10) |
| Daily stress x Collective eff. | 0.36 (0.05, 0.67)* | -0.18 (-0.51, 0.14) | -0.07 (-0.45, 0.30) | -0.06 (-0.89, 0.77) | 0.12 (-0.83, 1.07) | 0.42 (-0.48, 1.32) |

SD: Standard Deviation

^1^Estimated using general linear regression models, adjusting for participant age, sex, race/ethnicity, household income, parental education, parental marital status, and month. Because sleep variability measures were calculated at the participant level (i.e., by taking the person-specific standard deviation of each sleep variable across nights), models were at the participant rather than night level. Models included 148 participants with ≥2 nights of actigraphy data (needed to calculate variability measures).

*p-value <0.05

**Supplemental Table 6. Interactions of Stress and Neighborhood Social Environment Variables on Self-Reported Adolescent Sleep Outcomes**

| Interaction Term | Sleep Outcome Scores, β (95% CI)^1^ | | Within-Person Variability, β (95% CI)^1^ | |
| --- | --- | --- | --- | --- |
|  | Sleep Problems | Sleep Environment Disruptions | Sleep Problems SD | Sleep Environment Disruptions SD |
| PSS-10 Score x Safety | -0.01 (-0.02, 0.01) | -0.003 (-0.01, 0.01) | 0.002 (-0.01, 0.01) | -0.01 (-0.01, 0.003) |
| PSS-10 Score x Collective eff. | -0.001 (-0.02, 0.02) | -0.001 (-0.01, 0.01) | 0.006 (-0.01, 0.02) | -0.001 (-0.01, 0.01) |
| Daily stress score x Safety | -0.06 (-0.15, 0.03) | 0.004 (-0.05, 0.06) | -0.12 (-0.33, 0.08) | -0.01 (-0.15, 0.12) |
| Daily stress score x Collective eff. | -0.06 (-0.19, 0.08) | 0.01 (-0.07, 0.10) | -0.10 (-0.46, 0.26) | -0.02 (-0.25, 0.22) |
| Daily stress x Safety | -0.02 (-0.13, 0.09) | 0.01 (-0.05, 0.08) | -0.08 (-0.44, 0.28) | -0.02 (-0.29, 0.25) |
| Daily stress x Collective eff. | 0.05 (-0.11, 0.22) | 0.07 (-0.03, 0.17) | -0.21 (-0.76, 0.33) | -0.07 (-0.48, 0.34) |

SD: Standard Deviation

^1^Estimated using general linear regression models, adjusting for participant age, sex, race/ethnicity, household income, parental education, parental marital status, and month. Because sleep variability measures were calculated at the participant level (i.e., by taking the person-specific standard deviation of each sleep variable across nights), models were at the participant rather than night level. Models included 148 participants with ≥2 nights of actigraphy data (needed to calculate variability measures).

*p-value <0.05
